# Trends and correlates of unhealthy dieting behaviours among adolescents in the United States, 1999–2013

**DOI:** 10.1186/s12889-018-5348-2

**Published:** 2018-04-17

**Authors:** Sarah N. M. Chin, Anthony A. Laverty, Filippos T. Filippidis

**Affiliations:** 1 Department of Primary Care and Public Health, School of Public Health, Imperial College London, 310 Reynolds Building, St. Dunstan’s Road, London, W6 8RP UK; 2 Public Health Policy Evaluation Unit, School of Public Health, Imperial College London, London, UK

**Keywords:** Diet, Adolescents, United States, Vomiting, Fasting, Weight, Overweight, Obesity

## Abstract

**Background:**

The increase in adiposity problems among United States adolescents has been accompanied by persistently high prevalence of unhealthy dieting behaviours (UDBs) such as fasting, taking diet pills/powders/liquids, and vomiting/taking laxatives. This study aimed to examine the associations of self-perceptions of weight status, weight change intentions (WCIs) and UDBs with sex, age and race, as well as trends of UDBs in American adolescents across the weight spectrum.

**Methods:**

Data come from the biennial cross-sectional, school-based surveys, the Youth Risk Behaviour Surveillance System (1999–2013, *n* = 113,542). The outcome measures were the self-reported UDBs: fasting for 24 h or more; taking diet pills/powders/liquids; and vomiting/taking laxatives. Sex-stratified logistic regressions assessed relationships between weight status misperceptions across all weight statuses, race and WCIs with UDBs. Differential trends between races were assessed using race*year interaction terms.

**Results:**

In males, all non-White races had higher odds of fasting and vomiting/taking laxatives than Whites (except fasting in Hispanic/Latinos), with Adjusted Odds Ratios (AORs) between 1.44 and 2.07. In females, Black/African Americans and Hispanic/Latinos had lower odds of taking diet pills/powders/liquids compared to Whites (AORs 0.50 and 0.78 respectively). Racial disparities persisted throughout the study period. Prevalence of fasting and vomiting/taking laxatives did not change between 1999 and 2013 for all races, while taking diet pills/powders/liquids decreased. Compared to individuals of normal weight who were accurate weight status perceivers, individuals of almost all other combinations of weight status and weight status perception had significantly higher odds of displaying any UDB outcome. Overestimation of weight status was found to be the strongest determinant of UDBs. Compared to individuals endorsing "not wanting to do anything" about their weight, individuals endorsing all other WCIs (including wanting to gain weight) also showed significantly higher odds for every UDB outcome, with wanting to lose weight having AORs of the greatest magnitudes.

**Conclusions:**

Prevalence of UDBs is persistently high, and highest among females across all racial groups. UDBs may elevate undesired weight gain and weight loss in individuals who are obese/overweight and underweight respectively. Further research into weight status perceptions among adolescents may inform efforts to reduce UDBs.

## Background

Obesity has been steadily rising among adolescents in the United States (US) in the previous three decades with almost a fifth of all US youth aged 12–19 years now being obese [1]. Deviations from a healthy weight additionally include being underweight, which is also detrimental to health [2]. In the US prevalence of adolescent obesity, overweight, and underweight is an estimated 17.2%, 16.2%, and 3.8% respectively. Obesity is associated with metabolic syndrome and several cancers [3], while underweight is associated with higher rates of respiratory, intestinal, and reproductive problems [4]. Obesity has been found to be more prevalent among males and ethnic minorities, especially Black/African Americans and Hispanics/Latinos [1]. Meta-analyses suggest that current weight change programmes based on diet and exercise education vary in effectiveness and show high heterogeneity in results [5], with comprehensive behavioral family lifestyle interventions showing promising results [6]. Weight status perceptions are strong motivators of intention to lose weight among adults [7, 8] and weight status misperception is a stronger predictor of weight change intentions (WCIs) than actual body weight [9] among adolescents. It has been documented that adolescents who perceive themselves as overweight are more likely to report trying to lose weight and those who perceive themselves as underweight more likely to report trying to gain weight, regardless of their actual weight status [10].

The Centre for Disease Control and Prevention (CDC) estimate that close to one in two high school students in the US are trying to lose weight [11]. Intentions to lose weight are closely associated with unhealthy dieting behaviours (UDBs), including fasting or purging after meals via vomiting and taking laxatives [12]. UDBs have been suggested to be risk factors of several eating disorders (EDs), namely anorexia nervosa and bulimia nervosa, which result in undesirable weight loss [13, 14]. At the same time, individuals with obesity with the intention to lose weight are also more likely to attempt it through UDBs [15]. These UDBs are linked to potentially dangerous outcomes for individuals who are underweight, and are ineffective for long-term weight loss for persons with overweight [16, 17].

Hence monitoring trends and associations of UDBs over time is important to inform public health policies. The most recent data from the Youth Risk Behaviour Surveillance System (YRBSS) have not been used for this purpose [18, 19]. Additionally, the majority of studies have focused on people with overweight and obesity and so there is a paucity of literature analysing effects on underweight people [20, 21]. This study attempts to fill these gaps by using the latest YRBSS data to examine the associations of self-perceptions of weight, weight change intentions (WCIs) and UDBs with sex, age and race, as well as trends of UDBs in American adolescents across the weight spectrum.

## Methods

### Study design and population

We used data from the YRBSS, which is a biennial cross-sectional, school-based survey developed by the CDC in 1990. All survey cycles took place over a one-year period and results were published in June of the following year. It provides comparable health and health-risk behaviour data for a representative sample of American high schoolers [22]. The sample population includes all public and private schools with enrolled students in at least one of grades 9 to 12 in all 50 states of the US, and the District of Columbia. A more detailed account of the sampling methods is published on the CDC website [23]. All eight waves of the survey that included questions on UDBs (1999; 2001; 2003; 2005; 2007; 2009; 2011; and 2013) were analysed. The total sample size was *n* = 117,540 participants, with the smallest sample (*n* = 13,583) in 2013 and the largest (*n* = 16,410) in 2009.

Levels of missing data ranged from 0.35% for age to 2.71% for fasting and a final sample size of *n* = 113,542 was used in this analysis after excluding missing responses. All data were anonymized and publicly available; hence no ethical approval was required.

### Measures

#### Body mass index (BMI) and weight status

BMI was calculated using self-reported height and weight, then compared against the age- and sex-specific distribution of BMI in adolescence. Underweight was defined as a BMI < 5th percentile, normal weight as 5th ≤ BMI < 85th percentile, overweight as 85th ≤ BMI < 95th percentile, and obese as BMI ≥ 95th percentile [24].

#### Weight change intentions

WCIs were recorded using the item “Which of the following are you trying to do about your weight?”, and participants answered either “Lose weight”, “Gain weight”, “Stay the same weight”, or “I am not trying to do anything about my weight”. Binary variables of each option (Yes/No) were created and used as outcomes. Responses were also coded into a categorical explanatory variable for all UDB outcomes, with “I am not trying to do anything about my weight” as the reference category [25].

#### Unhealthy dieting behaviours

UDBs were assessed using 3 separate items “During the past 30 days, did you (i) go without eating for 24 hours or more (also called fasting) (ii) take any diet pills, powders, or liquids (PPLs) without a doctor's advice (iii) vomit or take laxatives, to lose weight or to keep from gaining weight?” [25]. Participants were required to respond Yes/No to each question.

#### Weight status misperceptions

Weight status perception was assessed by the question “How do you describe your weight?” and participants responded on a 5-point scale: very underweight, slightly underweight, about the right weight, slightly overweight, and very overweight [25]. Very and slightly underweight were grouped together as “Underweight”. We assessed if they had accurate perceptions (perceptions matching calculated weight status), or had weight misperceptions (discordance between perception and calculated weight status). Direction of misperceptions was operationalised as “overestimated” and “underestimated” if perceptions were higher or lower than calculated weight status respectively. A variable containing all 10 possible combinations of participant weight status (normal, underweight, overweight, obese) and weight status perception (accurate, underestimate, overestimate) was created to assess relationships across the spectrum of weight status perceptions. Healthy weight status with accurate perception was used as the reference category for this variable.

#### Socio-demographic variables

Participants also reported their age (14 years old or younger, 15, 16, 17, and 18 years old or older), sex (Male, Female) and race. Race was recorded as seven distinct categories in the survey, namely (i) White, (ii) Black/African American, (iii) Hispanic/Latino, (iv) American Indian/Alaska Native, (v) Asian, (vi) Native Hawaiian/Other Pacific Islander, and (vii) Multiple- Non-Hispanic [25]. The last four groups were collapsed into one group due to their small sample sizes, hence race was analysed in four categories (White, Black/African American, Hispanic/Latino, All other races).

### Statistical analyses

Logistic regression models were fitted to assess time trends of (i) overall UDBs, (ii) fasting, (iii) taking diet pills/powders/liquids and (iv) vomiting/taking laxatives (adjusted for age, race, WCIs, weight status perceptions and weight status). Effect modification by sex was identified and therefore, results are presented stratified by sex. Trends over time were analysed using the survey years as a linear time variable by using orthogonal coefficients which reflected the biennial spacing of the surveys; quadratic time variables were included in preliminary models but did not yield statistically significant results and were dropped from the final models. To examine potentially differential time trends between races, all logistic regression models contained an interaction term between race and calendar year. Adjusted Odds Ratios (AORs) for ‘year’ are interpreted as changes in the adjusted odds of reporting a UDB for each additional calendar year. Sampling weights provided by the CDC were used in order to account for an ethnic minority oversampling to compensate for nonresponse bias, and all reported proportions are weighted accordingly. All analyses were conducted with STATA 14.0.

## Results

The characteristics of the sample population are found in Table 1. The proportion of females overestimating their weight status was 21.9% in 1999 and 15.9% in 2013, while that of males was 6.9% in 1999 and 6.1% in 2013. Underestimation of weight status among females was 21.2% in 1999 and 25.0% in 2013, while in males was 30.6% in 1999 and 40.2% in 2013. Throughout the period assessed, 59%–63% of females and 26%–33% of males wanted to lose weight, while 6%–10% of females and 26%–30% of males wanted to gain weight.

**Table 1:** Sample socio-demographic characteristics

	Overall (*N* = 113, 542)	Females (*n* = 57, 602)	Males (*n* = 55, 940)
Weight (kg), *mean (SE)*	67.46 (0.126)	61.10 (0.118)	73.61 (0.158)
Height (m), *mean (SE)*	1.70 (0.0008)	1.63 (0.0007)	1.76 (0.0009)
BMI, *mean (SE)*	23.24 (0.037)	22.85 (0.044)	23.61 (0.042)
BMI percentile, *mean (SE)*	62.06 (0.198)	60.09 (0.237)	63.96 (0.221)
Race, *n (weighted %)*			
White	48,012 (60.6)	23,989 (59.9)	24,023 (61.2)
Black/African American	24,710 (14.0)	12,937 (14.4)	11,773 (13.7)
Hispanic/Latinos	30,697 (16.3)	15,555 (16.4)	15,142 (16.2)
All other races	10,123 (9.1)	5121 (9.3)	5002 (8.9)
Age, *n (weighted %)*			
≤ 14 years old	10,884 (11.1)	5994 (11.9)	4890 (10.3)
15 years old	25,651 (25.2)	13,415 (25.6)	12,236 (24.7)
16 years old	29,103 (26.3)	14,873 (26.0)	14,230 (26.5)
17 years old	29,527 (23.8)	14,880 (23.9)	14,647 (23.7)
≥ 18 years old	18,377 (13.7)	8440 (12.6)	9937 (14.8)
Weight Status, *n (weighted %)*			
Normal weight	73,765 (66.5)	39,267 (70.2)	34,498 (62.8)
Underweight	2803 (2.5)	1330 (2.4)	1473 (2.6)
Overweight	16,796 (14.2)	8568 (13.8)	8228 (14.7)
Obese	20,178 (16.8)	8437 (13.6)	11,741 (20.0)

### Unhealthy dieting Behaviours

The proportion of males performing at least one UDB to lose or maintain weight in the last 30 days was 10.4% in 1999 and 10.1% in 2013, while in females it was 26.2% in 1999 and 22.7% in 2013. In males, Black/African Americans were more likely to perform all UDBs except taking diet PPL compared to Whites (Table 2). Every other racial group also showed higher odds of vomiting/taking laxatives, and ‘All other races’ had higher odds of fasting compared to Whites (AOR 1.44, 95% CI 1.02–2.03). In females, however, the only racial disparities were in Black/African Americans and Hispanic/Latinos having lower odds of taking diet PPL compared to Whites (AOR 0.50, 95% CI 0.38–0.67 and AOR 0.78, 95% CI 0.63–0.96 respectively) (Table 3).

**Table 2:** AORs (95% CI) of race, weight status and perceptions, WCIs, and differential racial time trends for types of UDBs in Males

Category	n	Fasted for 24 h or more AOR (95% CI)	Took any diet PPL AOR (95% CI)	Vomited/Took laxatives AOR (95% CI)
Race				
White	23,676	ref	ref	ref
Black/African American	11,429	1.58 (1.30–1.92)	0.84 (0.60–1.18)	1.99 (1.42–2.78)
Hispanic/Latino	14,813	1.02 (0.83–1.26)	1.28 (0.87–1.89)	2.07 (1.36–3.16)
All other races	4891	1.44 (1.02–2.03)	0.93 (0.61–1.40)	2.05 (1.18–3.55)
Weight Status and Weight Status Perceptions				
Normal Weight; Accurate Perception	23,921	ref	ref	ref
Normal Weight; Underestimation	7661	1.69 (1.43–1.98)	1.05 (0.83–1.33)	2.27 (1.67–3.08)
Normal Weight; Overestimation	2511	1.49 (1.21–1.84)	1.71 (1.34–2.17)	3.46 (2.49–4.80)
Underweight; Accurate Perception	960	1.80 (1.26–2.56)	0.88 (0.52–1.47)	1.40 (0.79–2.47)
Underweight; Overestimation	510	2.64 (1.72–4.03)	1.05 (0.59–1.88)	3.01 (1.14–7.94)
Overweight; Accurate Perception	3213	0.82 (0.67–1.00)	1.26 (0.98–1.62)	1.27 (0.88–1.83)
Overweight; Underestimation	4749	1.26 (1.04–1.53)	1.91 (1.48–2.46)	1.83 (1.31–2.56)
Overweight; Overestimation	159	2.80 (1.69–4.64)	3.49 (1.76–6.93)	7.83 (3.62–16.94)
Obese; Accurate Perception	1532	1.53 (1.19–1.97)	3.38 (2.58–4.42)	5.93 (4.00–8.77)
Obese; Underestimation	10,174	1.41 (1.21–1.65)	2.31 (1.89–2.81)	2.39 (1.72–3.33)
WCI				
Do nothing about weight	11,521	ref	ref	ref
Lose weight	17,165	5.65 (4.73–6.76)	3.91 (3.16–4.85)	2.27 (1.66–3.09)
Gain weight	15,566	1.15 (0.95–1.40)	2.30 (1.77–3.00)	1.49 (1.04–2.14)
Stay the same weight	11,247	2.22 (1.86–2.64)	1.84 (1.43–2.37)	1.50 (1.15–1.95)
Year		0.99 (0.98–1.01)	0.96 (0.94–0.98)	0.98 (0.96–1.01)
Black/African American*Year		1.01 (0.98–1.03)	1.00 (0.97–1.02)	1.02 (0.98–1.06)
Hispanic/Latino*Year		1.02 (1.00–1.04)	1.02 (0.99–1.04)	0.99 (0.95–1.03)
All other races*Year		1.01 (0.97–1.04)	0.99 (0.96–1.03)	1.03 (0.99–1.08)

Note: All models are adjusted for age. *Significant AORs (*p*-value< 0.05)

**Table 3:** AORs (95% CI) race, weight status and perceptions, WCIs, and differential racial time trends for types of UDBs in Females

Category	n	Fasted for 24 h or more AOR (95% CI)	Took any diet PPL AOR (95% CI)	Vomited/Took laxatives AOR (95% CI)
Race				
White	23,717	ref	ref	ref
Black/African American	12,681	0.99 (0.83–1.17)	0.50 (0.38–0.67)	0.88 (0.59–1.32)
Hispanic/Latino	15,318	0.96 (0.80–1.15)	0.78 (0.63–0.96)	1.01 (0.77–1.32)
All other races	5023	1.09 (0.77–1.56)	0.88 (0.64–1.20)	1.42 (0.83–2.46)
Weight Status and Weight Status Perceptions				
Normal Weight; Accurate Perception	25,603	ref	ref	ref
Normal Weight; Underestimation	4895	2.00 (1.69–2.35)	1.48 (1.14–1.93)	1.96 (1.45–2.65)
Normal Weight; Overestimation	8414	1.66 (1.52–1.81)	1.75 (1.55–1.96)	1.94 (1.65–2.29)
Underweight; Accurate Perception	849	2.03 (1.46–2.82)	0.87 (0.45–1.68)	1.14 (0.57–2.29)
Underweight; Overestimation	483	2.07 (1.38–3.10)	1.62 (0.73–3.57)	1.21 (0.65–2.27)
Overweight; Accurate Perception	5391	1.24 (1.11–1.38)	1.86 (1.60–2.16)	1.41 (1.20–1.66)
Overweight; Underestimation	2503	1.32 (1.11–1.57)	1.28 (1.02–1.62)	1.16 (0.89–1.50)
Overweight; Overestimation	638	2.07 (1.59–2.70)	2.81 (1.98–3.99)	3.70 (2.50–5.46)
Obese; Accurate Perception	1880	1.55 (1.33–1.82)	2.83 (2.42–3.32)	2.16 (1.76–2.65)
Obese; Underestimation	6554	1.52 (1.35–1.71)	1.99 (1.71–2.30)	1.67 (1.37–2.04)
WCI				
Do nothing about weight	8546	ref	ref	ref
Lose weight	34,097	8.48 (7.24–9.93)	7.89 (6.04–10.31)	9.28 (7.09–12.14)
Gain weight	4621	1.34 (1.02–1.75)	2.04 (1.17–3.55)	3.00 (1.58–5.70)
Stay the same weight	10,042	2.02 (1.68–2.41)	1.49 (1.06–2.09)	1.89 (1.34–2.67)
Year		0.99 (0.98–1.01)	0.93 (0.91–0.95)	0.98 (0.96–1.00)
Black/African American*Year		1.00 (0.98–1.02)	0.99 (0.97–1.02)	1.01 (0.98–1.05)
Hispanic/Latino*Year		1.02 (1.00–1.04)	1.01 (0.99–1.03)	1.04 (1.01–1.06)
All other races*Year		0.99 (0.95–1.02)	0.99 (0.95–1.02)	1.00 (0.96–1.04)

Note: All models are adjusted for age. *Significant AORs (*p*-value< 0.05)

After stratifying by sex and adjusting for age, race, and weight status misperceptions by weight status, associations of WCIs with UDBs were explored. Compared with not trying to do anything about weight, every other WCI showed significantly higher odds for almost all UDB outcomes, with wanting to lose weight having the highest AORs. This ranged from AOR 2.27 (95% CI 1.66–3.09) for vomiting/taking laxatives in males (Table 2) to AOR 9.28 (95% CI 7.09–12.14) for vomiting/taking laxatives in females (Table 3). The only exception was for fasting in males who wanted to gain weight, which showed no statistically significant difference in odds to the WCI reference category (Table 2).

Compared with normal weight status participants who had an accurate perception of their weight status, most other categories were more likely to have performed an UDB the past 30 days among both males and females. Individuals with overweight and obesity and those who overestimated their weight status were in general the most likely to have fasted, vomited/taken laxatives or taken any diet PPL (Tables 2 and 3).

### Time trends

Racial differential time trends were varied for the different UDB outcomes. For both sexes, fasting and vomiting/taking laxatives showed no statistically significant time trends across all races. Taking diet PPL in both sexes exhibited statistically significantly decreasing trends (Males: AOR 0.96, 95% CI 0.94–0.98; Females: AOR 0.93, 95% CI 0.91–0.95 per calendar year) (Tables 2 and 3) and there were no statistically significant racial differences in the slope of this decline. Although the overall decline for vomiting/taking laxatives in females was not statistically significant, Hispanic/Latino females had a statistically significantly gentler decline (AOR 1.04, *p*-value for difference 0.006).

## Discussion

This nationally representative study has found that prevalence of Unhealthy Dieting Behaviours is persistently high among US adolescents and was highest among females across all racial groups. Types of UDBs differed by racial groups, although the prevalence of overall UDBs were comparable across the races, and there was a lack of significant differential time trends among races. These findings are largely consistent with other large scale adolescent studies and support current literature in highlighting the sex and racial differences in UDBs [16]. These differences are of concern as UDBs are associated with undesired weight gain and weight loss in individuals with obesity/overweight and underweight respectively, thus exacerbating weight mismanagement. This study also showed that weight status underestimation and desire to gain weight were not protective against UDBs when compared to normal weight accurate weight status perceivers who did not want to do anything about their weight.

UDBs paradoxically lead to further weight gain as short-term UDBs may divert adolescents away from more effective long-term weight management practices [16, 17]. It has been hypothesised that the weight gain is due to the association of UDBs with ineffective weight-loss behaviours [16]. These include skipping breakfast, decreasing fruit and vegetable consumption, and reducing physical activity, all of which have been shown to be linked to weight gain [26–28]. This might be particularly detrimental to adolescents who are overweight since further weight gain may trigger even more UDB practices if desire to lose weight intensifies. As UDBs are predictors of both EDs and weight loss/gain, they are potentially detrimental to adolescents who are both underweight or obese/overweight.

This analysis also found significant racial disparities in UDBs. The generally higher socioeconomic status (SES) of Whites [29] may provide them with greater access to diet PPL than other races due to the cost of those products, compared to fasting and vomiting which involve no monetary cost. These UDB disparities are troubling since fasting and purging behaviours have been reported to be important precursors of anorexia nervosa and bulimia nervosa respectively [30]. Cultural beliefs and body images in different cultures may also be a factor, although we found no racial disparities in the overall prevalence of UDBs. We found that there were no racially differential time trends in UDBs from 1999 to 2013, with the exception of Hispanic/Latino females who had worsening trends in vomiting/taking laxatives compared to Whites. The lack of significant differential time trends among races indicates a persistence in racial disparities, which predominantly disadvantage minority groups.

The association between wanting to lose weight and UDBs was much stronger in females than in males, which may indicate a difference in the magnitude of the impact that weight loss intentions have between sexes. Interestingly, we found that with the exception of fasting in males, all those who wanted to gain weight also displayed higher odds of UDBs compared to the WCI referent. This could be due to adolescent males wanting to gain muscle mass but lose fat mass, resulting in them participating in UDBs to lose fat despite wanting to gain weight overall. However, this result requires further investigation. Overall though, it is consistent with the message that those not intending to do anything about weight are the least likely to exhibit UDBs.

Prior research has shown that in adolescents with overweight/obesity weight loss attempts are less likely among those who underestimate their weight status, compared to those with accurate perceptions [31, 32]. Underestimating the weight status has also been found to be protective against a number of disordered eating behaviours among adolescents with overweight/obesity [33]. Our study included individuals across the weight spectrum and we have further adjusted for WCIs; hence, similar direct comparisons within the group of adolescents with overweight/obesity are not straightforward. However, we found that the desire to lose weight was associated with all UDBs in both males and females, which may highlight a pathway that could explain both our findings and those from the studies mentioned above.

### Implications for research and practice

Taking the implications of this study’s findings on the whole, targeting UDBs by tackling the high weight status misperception and inappropriate WCI levels could mitigate weight mismanagement among US adolescents. Thus, public health recommendations could include providing quality health education in schools regarding what accurate definitions of normal weight are. This would be essential in abating weight misperception [34]. However, systemic factors may affect an individual’s weight status perception and WCI. Social environmental factors such as media depiction of normal weight can lead to negative body image and weight obsession. Such issues could potentially be addressed though fostering school environments that discourage comparisons and teasing of body shapes and sizes [35]. The aim of public health interventions to alleviate weight mismanagement can be assisted by reducing UDBs.

Additionally, the persistently high prevalence of UDBs among adolescents may warrant more focus on strategies explicitly aiming to reduce UDBs on top of the current practice of promoting healthy dieting or exercise regimes. To ensure proper weight control techniques, weight management programmes could also warn adolescents about the ineffective and harmful consequences of UDB practices.

### Strengths and limitations

This is the first national study to examine weight status misperceptions, WCIs and UDBs among US adolescents across the weight spectrum. We used a large representative sample of the US student population across 14 years; however, the cross-sectional nature of the study precludes us from making causal inferences. Longitudinal research would be needed to elucidate reasons behind observed correlates and trends. Additionally, YRBSS uses self-reported height and weight which has been shown to lead to an underestimation of obesity prevalence in Europe [36, 37] and the US [38] by up to 12 percentage points. This might thereby bring in misclassification bias by underestimating weight status misperceptions as well. UDB data were also collected via self-reporting which might result in an over or under-reporting of UDBs. However, the YRBSS has undergone multiple test-retest reliability assessments and shown acceptable levels of validity [39]. Finally, the YRBSS lacks some socio-demographic variables that might influence the strength of the association and trends found in this study. Such variables include the students’ SES and parental income, both of which have been found to influence obesity rates and weight status perception [40].

## Conclusion

This study found weight status misperceptions in adolescents across all weight statuses and WCIs to be associated with higher UDBs. Apart from a slight decline in diet PPL use, these behaviours have been persistent between 1999 and 2013. Racial disparities in the prevalence of different UDB types were also observed, with no improvements over time. As UDBs have been previously established to increase undesired weight gain and weight loss in individuals who are obese/overweight and underweight respectively, its reduction is likely to address weight mismanagement at both ends. Hence, current trends are concerning, and concerted action will be needed on a number of levels. Public health policies might aim for adolescents to improve weight status perception accuracy to potentially reduce inappropriate weight preoccupation and subsequently UDBs.

## Abbreviations

AOR: Adjusted Odds Ratio
BMI: Body Mass Index
CDC: Centres for Disease Control and Prevention
CI: Confidence Interval
ED: Eating disorders
PPL: Pills/powders/liquids
SES: Socioeconomic status
UDB: Unhealthy dieting behaviour
US: United States
WCI: Weight chage intention
YRBSS: Youth Risk Behaviour Surveillance System
